# Reduced Native T1 Values of Wrist Tissues in Transthyretin Cardiac Amyloidosis

**DOI:** 10.3390/jcm14207374

**Published:** 2025-10-18

**Authors:** Jean-François Deux, Pierre Brugières, Mounira Kharoubi, Amira Zaroui, Silvia Oghina, Thibaud Damy, Ruxandra Cosson

**Affiliations:** 1 Division of Radiology, Diagnostic Department Geneva University Hospitals, 1205 Geneva, Switzerland; 2 Department of Radiology, Henri Mondor Hospital, 94010 Creteil, France; brugieres.pierre@gmail.com (P.B.); ruxandra.cosson@aphp.fr (R.C.); 3 Department of Cardiology, Referral Center for Cardiac Amyloidosis, Filiere Cardiogen, GRC Amyloid Research Institute all at APHP CHU Henri Mondor, 94010 Creteil, France; mounira.kharoubi@aphp.fr (M.K.); amira.zaroui@aphp.fr (A.Z.); silvia.oghina@aphp.fr (S.O.); thibaud.damy@aphp.fr (T.D.); 4 Department of Radiology, Hôpital Privé Paul d’Egine, 94400 Champigny-sur-Marne, France

**Keywords:** wrist, Transthyretin amyloidosis, MRI, native T1 mapping, cardiac amyloidosis

## Abstract

**Background/Objectives:** Carpal tunnel syndrome (CTS) may signal extracardiac amyloid deposition years before transthyretin cardiac amyloidosis (ATTR-CA). This study investigated potential alterations of wrist tissue T1 values in ATTR-CA patients. **Methods:** Patients with ATTR-CA and healthy volunteers underwent 1.5T wrist MRI using a gradient echo sequence. Manual contouring of the transverse carpal ligament (TCL), median nerve (MN), sheaths of the flexor carpi tendons (SFCT), subcutaneous fat (SCF), muscle of the thenar eminence (MTE), and global wrist (GCW) was performed by two readers. Native T1 values were compared between groups. **Results:** Thirty-six patients with ATTR-CA (mean age, 78 ± 9 years; 32 men) and 69 volunteers (43 ± 14 years; 24 men) were evaluated. Mean native T1 values of TCL, MN, SFCT, SCF, and GCW were significantly lower in patients than in volunteers (*p* < 0.005 for all). Multivariable regression adjusted for age and sex confirmed these associations. SCF T1 was significantly lower in patients with CTS symptoms (885 [762–1080] ms) than in asymptomatic patients (1041 [949–1267] ms, *p* = 0.04). The highest area under the curve (AUC) for detecting CA was obtained for SFCT (AUC = 0.85; 95% CI 0.77–0.93). **Conclusions:** Patients with transthyretin cardiac amyloidosis show a significant reduction in the native T1 of wrist tissues compared with controls. These preliminary findings suggest that wrist T1 mapping may serve as a non-invasive marker of peripheral amyloid involvement, but require further validation in larger, age-matched, histologically validated studies.

## 1. Introduction

Transthyretin cardiac amyloidosis (ATTR-CA) is a well-recognized cause of heart failure with preserved ejection fraction (HFpEF) [1,2]. It results from the extracellular deposition of amyloid fibrils in the myocardium, leading to progressive myocardial stiffening and often concentric thickening of the ventricular walls [3,4]. Cardiac MRI plays a central role in the non-invasive diagnosis of CA, with parametric mapping techniques such as native T1 and extracellular volume (ECV) mapping providing quantitative markers of myocardial infiltration [5,6,7,8].

Over the last decade, the awareness and diagnostic rates of ATTR-CA have markedly increased, driven by advances in imaging and the availability of disease-modifying therapies. Beyond the heart, extracardiac manifestations have gained growing attention as potential early warning signs of systemic amyloid deposition [9,10]. Among them, carpal tunnel syndrome (CTS) is now recognized as one of the most frequent and clinically relevant extracardiac clues to ATTR amyloidosis, often preceding cardiac involvement by several years and showing a strong epidemiological association with the disease [11,12,13,14,15]. To improve early detection of ATTR-CA, some authors have proposed performing tenosynovial biopsies during carpal tunnel release surgery to identify amyloid deposits [16,17,18]. While promising, this approach remains invasive and requires specific histological expertise.

MRI could be an attractive technique to non-invasively detect extracardiac amyloid deposits using T1 mapping. Authors have reported a reduction in native T1 in the liver [19,20] and an increase in ECV in the liver and the spleen [21,22] in patients with amyloidosis, but to our knowledge, no prior study has explored the value of wrist MRI T1 mapping in detecting amyloid deposition in patients with ATTR-CA. Intra-articular masses, synovitis, and cystic bone lesions have been reported in dialysis-related amyloidosis using wrist MRI [23,24], but this form of amyloidosis is rare, its pathophysiology differs from ATTR, and T1 mapping images were not acquired.

We hypothesized that ATTR-CA patients with CTS may exhibit local amyloid infiltration at the wrist, detectable as alterations in native T1 values of specific anatomical structures. Therefore, the aim of this study was to evaluate native T1 values in six predefined wrist structures in ATTR-CA patients (with and without CTS) and in healthy controls, in order to assess the diagnostic performance of peripheral T1 mapping for detecting amyloid involvement.

## 2. Materials and Methods

### 2.1. Study Population

**Figure 1:**
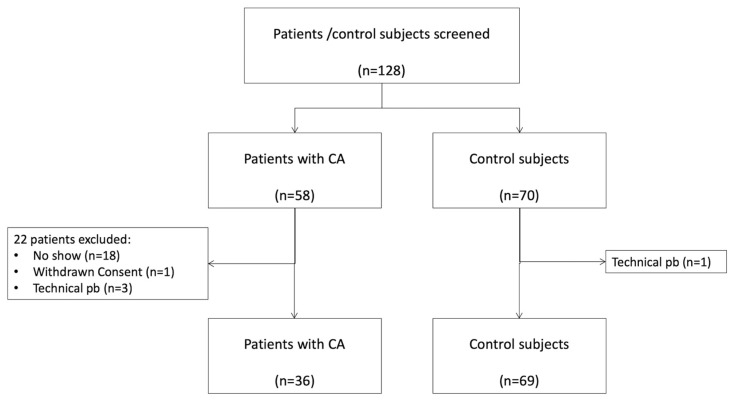
Patient flowchart.

This prospective study (Trial registration number: NCT05150353) was approved by the ethics committee, and informed consent for participation in this research was obtained from all participants and volunteers. Fifty-eight consecutive patients with ATTR-CA were considered for inclusion between 24 January 2020 and 24 April 2022. Inclusion criteria were age over 18 years and evidence of cardiac amyloidosis by imaging or cardiac biopsy. Exclusion criteria were a history of both wrist surgery and a known systemic disease that could affect the wrist. After applications of the criteria of inclusion, 36 patients were finally included: 31 participants with wild-type TTR and 5 participants with variant TTR. The diagnosis of ATTR-CA was confirmed in all patients by strong cardiac fixation on bone scintigraphy in the absence of gammopathy or endomyocardial biopsy. Figure 1 shows the patient flowchart. Sixty-nine volunteers without clinical symptoms or medical history were also included.

### 2.2. Data Collection

For each patient and control subject, the following data were collected: age, sex, body mass index, the presence of diabetes, hypertension, and dyslipidemia, the presence of symptoms suggestive of CTS, and, for patients, possible treatment with tafamidis. The diagnosis of CTS was based on clinical symptoms such as nocturnal paresthesia, pain, or weakness in the median nerve territory; neurophysiological testing was not systematically performed. Biological data (Troponin T-HS level, NT pro-BNP level, CRP level, and blood creatinine level) and imaging data from echocardiography, cardiac magnetic resonance, and cardiac scintigraphy were also collected in patients with CA. All these data are reported in Table 1.

### 2.3. Image Acquisition

**Figure 2:**
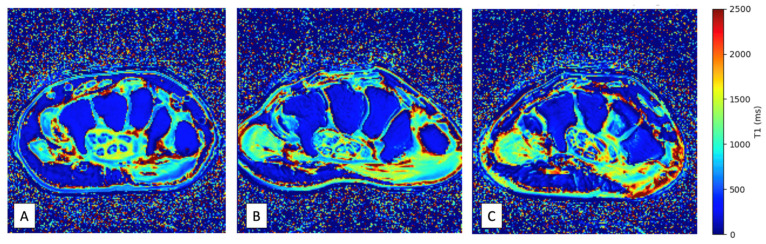
Examples of T1 mapping wrist images obtained in ATTR-CA patients with (**A**) and without (**B**) CTS, and in a healthy volunteer (**C**).

All MRI examinations were performed at two different sites, each equipped with an identical 1.5-T imaging system (AVANTO; Siemens Healthcare, Erlangen, Germany), featuring an 8-channel phased-array coil, and followed the same imaging protocol. The wrist was examined in a pronated position with the arm adducted over the head and the fingers extended. The right wrist was selected for all asymptomatic patients and for all volunteers. In patients with wrist pain suspicious for CTS, the painful side was chosen (in the case of bilateral symptoms, the most painful side was chosen). Axial MRI images at 3 levels of the wrist (proximal, middle and distal levels) were obtained from the distal radioulnar joint proximally to the base of the metacarpals distally using a gradient echo sequence acquired 6 times at the same level using increasing flip angles (2, 5, 7, 10, 12 and 15 degrees) with the following parameters: TR: 5.81 ms, TE: 2.18 ms; 8 averages, BW: 390 MHz, FOV: 90 × 90 mm; matrix 192 × 154; pixel size 0.5 × 0.6 mm; slice thickness 1.7 mm, acquisition time: 57 s. T1 mapping images (Figure 2) of the wrist were reconstructed from gradient echo images using a previously reported method [23]. A proton density spin echo sequence with fat saturation was also acquired at the same level with the following parameters: TR: 2000 ms, TE: 31 ms; flip angle: 180 degrees. BW: 150 MHz, FOV: 90 × 90 mm; matrix 320 × 320; pixel size 0.3 × 0.3 mm; slice thickness 3 mm, acquisition time: 2 min 06 s. The total MRI scan time was approximately 10 min.

### 2.4. Image Analysis

**Figure 3:**
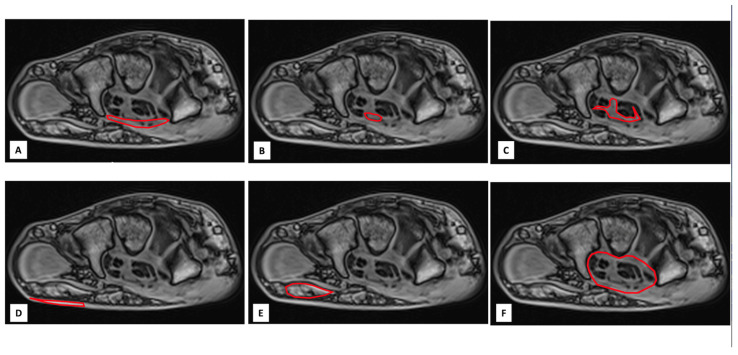
Examples of manually contouring (red line) of transverse carpal ligament (TCL) (**A**), median nerve (MN) (**B**), sheaths of the flexor carpi tendons (SFCT) (**C**), subcutaneous fat of the wrist (SCF) (**D**) and muscle of thenar eminence (MTE) (**E**) at the proximal level of the wrist on axial gradient echo images. The sixth measure (**F**) consisted of a global contouring of the wrist (GCW) encompassing all the above-mentioned structures.

Image analysis was performed by consensus of 2 authors (RC, JFD). A visual analysis of the MRI images, looking for an abnormal mass at the level of the carpus, suggesting amyloid deposits, was first performed by the authors. In a second step, the following 5 anatomical structures of the wrist, classically involved in CTS, were manually contoured by consensus of 2 authors (RC, JFD) from axial 2 degrees flip angle gradient echo images at 3 levels of the wrist (proximal, middle and distal levels): the transverse carpal ligament (TCL), the median nerve (MN), the sheaths of the flexor carpi tendons (SFCT), the subcutaneous fat of the wrist (SCF) and the muscle of the thenar eminence (MTE). A sixth measurement consisting of a global contouring of the wrist (GCW) encompassing all the above-mentioned structures was also performed (Figure 3). The 6 contours for each slice level were then copied onto the gradient echo images acquired with the other flip angles. Manual adjustment of the contours was performed in case of a shift between the images. In case of difficulties in finding the boundaries of the above-mentioned structures on gradient echo images, the operators used the images of the proton density spin echo sequence. The signal intensities (SI) of all analyzed structures were noted for each flip angle and for each measurement plane. The SI values obtained at the 3 levels of the wrist were averaged. Thus, 6 mean SI values (one for each flip angle) were obtained for each structure. The native T1 value of each structure was then calculated from these 6 values using a previously reported method [25].

### 2.5. Statistical Analysis

Categorical variables were described as numbers (percentages) and continuous variables as mean ± standard deviation (SD) or median (interquartile range) according to their distribution. Differences between groups were determined by the Wilcoxon rank sum test for continuous variables, and χ^2^ test or Fisher’s exact test for categorical variables. Multivariable linear regression analysis was performed to account for potential confounder variables (age and sex), with T1 values of each wrist structure as dependent variables. Pearson correlation coefficients were calculated to assess the correlation between continuous variables. A correlation with a Pearson coefficient greater than 0.5 was considered strong, between 0.3 and 0.5 moderate, and below 0.3 weak. Receiver operating characteristic (ROC) analysis was applied to T1 values of the wrist structures to detect patients with CA. Reliability of MR measurements was assessed by the same two readers for a random sample of 20 patients, by computing intraclass correlation coefficients (ICCs) and mean bias with 95% limits of agreement according to the Bland–Altman method. ICC values < 0.5 indicate poor reliability, between 0.5 and 0.75 moderate reliability, between 0.75 and 0.9 good reliability, and >0.90 excellent reliability. A *p*-value ≤ 0.05 was considered significant. Statistical analysis was conducted using SPSS (version 16.0).

## 3. Results

### 3.1. Population Characteristics

Thirty-six patients with CA (mean age: 78 ± 9; 32 men) and 69 volunteers (mean age: 43 ± 14; 24 men) were included in this study. Patients with CA were significantly older and more likely to be male than were volunteers. Sixteen patients with CA (44%) had symptoms of CTS, with bilateral involvement in 12 patients (75%). All clinical, biological, and imaging data are reported in Table 1.

### 3.2. Image Analysis

**Figure 4:**
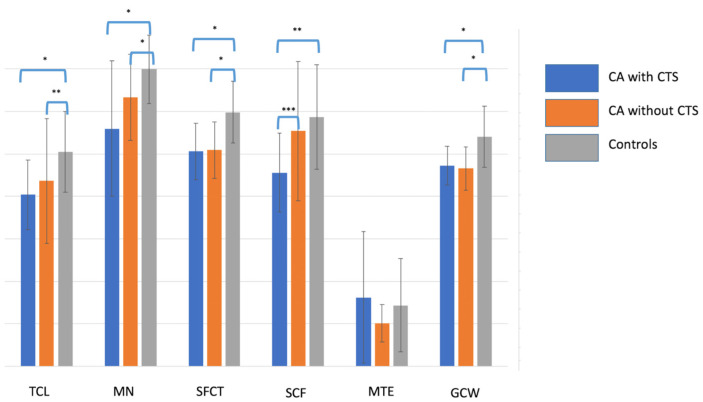
Histogram graph showing the T1 values of the transverse carpal ligament (TCL), median nerve (MN), sheaths of the flexor carpi tendons (SFCT), subcutaneous fat of the wrist (SCF), muscle of thenar eminence (MTE) and global contouring of the wrist (GCW) in patients (CA) with and without carpal tunnel syndrome (CTS) and in control patients. *: *p* < 0.001; **: *p* < 0.005; ***: *p* < 0.05.

No macroscopic masses suggestive of amyloid deposits were identified in the carpal tissues of patients or control subjects. Mean native T1 values of TCL, MN, SFCT, SCF, and GCW were all significantly lower in patients with CA compared with controls (*p* < 0.005 for all, Table 2). Importantly, these differences persisted when the analysis was restricted to controls older than 45 years (Table S1) and remained significant after multivariable adjustment for age and sex, confirming that T1 values of these structures were independent determinants of amyloid status. Neither age nor sex showed any significant association with amyloid status (*p* > 0.05 for both). Finally, the native T1 value of SCF was significantly lower in patients with CTS than in asymptomatic patients (885 [762–1080] ms vs. 1041 [949–1267] ms, *p* = 0.04; Figure 4).

### 3.3. Receiver Operating Characteristic Curve Analysis

**Figure 5:**
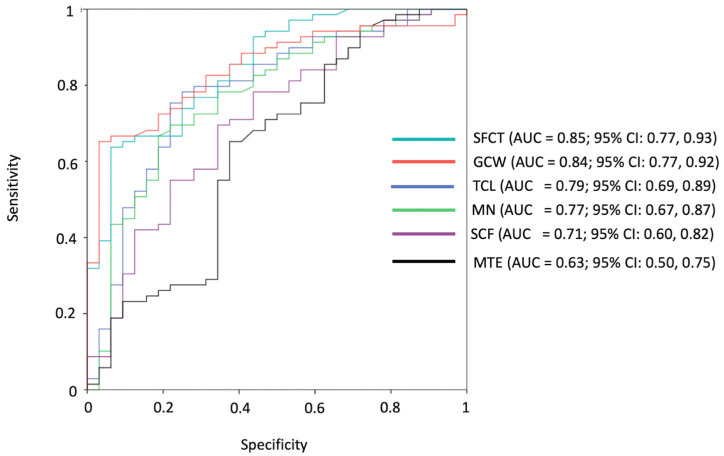
ROC curves of transverse carpal ligament (TCL), median nerve (MN), sheaths of the flexor carpi tendons (SFCT), subcutaneous fat of the wrist (SCF), muscle of the thenar eminence (MTE), and global contouring of the wrist (GCW) to detect patients with cardiac amyloidosis.

The highest areas under the curve (AUCs) for patients diagnosed with CA as opposed to control subjects were obtained for SFCT (AUC = 0.85; 95% CI: 0.77, 0.93), GCW (AUC = 0.84; 95% CI: 0.77, 0.92), and TCL (AUC = 0.79; 95% CI: 0.69, 0.89). For SFCT, a T1 value lower than 1149 ms achieved a sensitivity of 92% (33 of 36 patients) and a specificity of 65% (45 of 69 control subjects) in detecting patients with CA. All ROC curves are reported in Figure 5.

### 3.4. Correlations with Clinical, Biological, and Imaging Parameters

Mean T1 values of TCL, MN, SFCT, and GCW were weakly correlated with age (r = −0.25, r = −0.39, r = −0.42, and PCC = −0.38, respectively, for TCL, MN, SFCT, and GCW; *p* < 0.01 for all). No correlations were detected between T1 values and biological and radiological registered parameters except for left atrial volume, which was significantly correlated with T1 value of MN (r = −0.58, *p* < 0.02), SFCT (r = −0.64, *p* < 0.01), and GCW (r = −0.63, *p* < 0.01). No significant correlation was found between mean T1 values of the wrist structures in patients with CTS and biological or imaging parameters, except between left atrial volume and T1 value of MN (r = −0.85, *p* = 0.03).

### 3.5. Reproducibility

The ICCs were 0.859 [95% confidence interval (CI), 0.512–0.960], 0.970 [95% CI, 0.889–0.992], 0.924 [95% CI, 0.750–0.977], 0.829 [95% CI, 0.310–0.957], 0.741 [95% CI, 0.380–0.930] and 0.967 [95% CI, 0.886–0.981] for T1 measurement of TCL, MN, SFCT, SCF, MTE, and GCW, respectively.

## 4. Discussion

In this study, we demonstrate that patients with transthyretin cardiac amyloidosis (ATTR-CA) exhibit significantly reduced native T1 values in several anatomical structures of the wrist, which are typically involved in amyloidosis, compared with healthy controls. This reduction remained significant after adjustment for age and sex. The native T1 value of SCF was significantly lower in patients with CTS than in asymptomatic patients, which may suggest a link with amyloid burden. To the best of our knowledge, this is the first study reporting alterations in native T1 values in wrist tissues in patients with ATTR-CA. Overall, these results should be interpreted as preliminary, hypothesis-generating observations that require validation in larger, histologically confirmed cohorts.

Despite the lack of histological confirmation, the observed T1 differences between patients and controls could plausibly be related to amyloid deposition. This interpretation is supported by several findings: the regions of interest were selected in anatomical sites typically affected by amyloid infiltration; one analyzed structure (SCF) showed significantly lower values in CTS patients compared with asymptomatic individuals, suggesting a higher local amyloid burden in symptomatic cases; and a trend toward a stepwise reduction in T1 values across most structures was observed, progressing from controls to asymptomatic patients and then to patients with carpal tunnel syndrome (CTS) (Figure 3). Furthermore, statistical analyses indicated that these T1 differences were not explained by age or sex. Although these results are consistent with the hypothesis of progressive peripheral amyloid involvement, they should be regarded as preliminary, pending histopathological validation in future studies.

The paradoxical T1 reduction observed in our study contrasts with the increase typically reported in cardiac [6] or hepatic [20] amyloidosis, where interstitial expansion leads to elevated native T1 values. Several structural and biochemical studies have demonstrated that amyloid fibrils can interact with lipids and apolipoproteins, forming lipid–protein complexes or lipid-enriched fibrils that modify local magnetic properties [26,27,28,29]. Lipid enrichment within amyloid deposits has been confirmed in both familial and dialysis-related amyloidosis, where lipid droplets are embedded in fibrillar aggregates [27]. The “lipid-chaperon” hypothesis further suggests that lipids can modulate amyloid conformation and local microenvironmental organization, potentially influencing proton mobility and relaxation behavior [30]. Because lipid protons exhibit inherently short T1 relaxation times, even small lipid fractions or partial-volume effects could decrease the apparent T1 of affected tissues. The wrist, being rich in connective and adipose components, may thus display this paradoxical T1 shortening. Collectively, these findings support the idea that reduced T1 values may reflect both amyloid infiltration and lipid-related compositional changes within peritendinous and subcutaneous tissues.

Only limited correlations were found between wrist T1 values and cardiac imaging or biomarker data, with the exception of left atrial diastolic volume, a recognized marker of diastolic dysfunction [31,32] and an indirect marker of amyloid burden [33,34]. The lack of broader correlations may reflect the modest sample size of this study or the possibility that cardiac and extracardiac amyloid deposition are not directly related, as has been demonstrated in hemochromatosis, where cardiac and hepatic iron overload can occur independently [35,36].

The reliability of the T1 measurements obtained in this study was generally excellent. Measurements were performed directly on native images, with manual adjustment of regions of interest (ROIs), rather than on parametric T1 maps. This approach was chosen to maximize anatomical precision, particularly given the small size and complex geometry of certain wrist structures, which can make accurate delineation on T1 maps challenging. Interestingly, the global carpal wrist (GCW) contour, a more holistic and less labor-intensive parameter, demonstrated diagnostic performance nearly equivalent to that of the SFCT measurement—the most discriminant local structure. This finding suggests that GCW could serve as a practical and reproducible alternative to fine manual delineation in clinical applications.

Neurophysiological assessment of the median nerve was not systematically available in this cohort, as the study primarily focused on MRI-based tissue characterization rather than functional evaluation. Nevertheless, the observation of lower T1 values in patients with clinically manifest CTS supports a potential relationship between tissue changes and nerve dysfunction. Integrating electrophysiological parameters in future studies would help determine whether T1 reduction scales with the degree of conduction impairment, thereby strengthening the link between amyloid infiltration and functional nerve involvement.

Contrary to previous reports in dialysis-related amyloidosis [24,37], we did not observe visible amyloid deposits or erosive changes on visual inspection of MR images. This likely reflects differences in pathophysiology between ATTR and β2-microglobulin amyloidosis. The latter is known to produce large intra-articular masses and bone erosions, whereas ATTR tends to induce subtler peritendinous deposits without destructive features, rather than large nodular deposits.

This study has several limitations. First, patients with cardiac amyloidosis were significantly older than controls, which represents a potential bias since native T1 values may vary with age. Nevertheless, all analyses were adjusted for age and sex, and the observed group differences remained statistically significant, suggesting that age alone does not account for the observed T1 reductions. Another limitation is the absence of a control group with non-amyloid carpal tunnel syndrome. This limitation prevents determining whether the observed T1 reduction is specific to amyloid infiltration or could also occur in other forms of CTS related to mechanical or inflammatory causes. Future studies including such control groups are warranted to assess the specificity of peripheral T1-mapping findings. Finally, the patients studied did not undergo wrist surgery to confirm the diagnosis of CTS, and we have no histological evidence of amyloid deposition in the analyzed structures.

In conclusion, our results indicate that ATTR-CA patients show reduced native T1 values in wrist structures commonly involved in CTS, supporting the hypothesis of peripheral amyloid infiltration. These findings should, however, be considered preliminary and exploratory, pending validation in larger, age- and sex-matched cohorts, including non-amyloid CTS comparators and histopathological confirmation. If confirmed, global wrist T1 mapping could represent a simple, reproducible parameter with potential clinical application as a screening tool in idiopathic or bilateral CTS, complementing conventional histological approaches.

## Figures and Tables

**Table 1:** Characteristics of patients with cardiac amyloidosis (CA) and control subjects.

	CA (n = 36)	Controls (n = 69)	*p*
Males	32 (89)	24 (35)	<0.001
Age (y)	78 ± 9	43 ± 14	<0.001
Body mass index (kg/m^2^)	25 ± 4	26 ± 4	0.89
**Cardiovascular risk factors**			
Diabetes	7 (19)	0	<0.001
Hypertension	26 (72)	0	<0.001
Dyslipidemia	12 (33)	0	<0.001
NYHA class: I, II, III, IV, n	8/20/6/2	69/0/0/0	
Carpal tunnel syndrome	16 (44)	0	<0.001
Surgery for carpal tunnel syndrome *	7 (19)	0	<0.001
Treatment with tafamidis	8 (22)	0	<0.001
**Laboratory parameters**			
Troponin us (ng/L)	47 (31–66)		
NT-pro BNP (ng/L)	3140 (605–3170)		
CRP (mg/L)	3.1 (0.6–5.6)		
Blood creatinine (μmol/L)	95 (81–111)		
**Echocardiography**			
LVEF (%)	49 ± 12		
IVS thickness (mm)	17 ± 3		
Left atrial volume (mL/m^2^)	49 ± 12		
**Cardiac MRI**			
LVEF (%)	51 ± 11		
LV mass (g/m^2^)	78 ± 23		
Native myocardial T1 (ms)	1115 ± 76		
ECV (%)	47 ± 10		
**Cardiac scintigraphy**			
Grade of uptake	2.9 ± 0.5		
Ratio Heart/Mediastinum	1.4 ± 0.3		

Data are median (%), mean ± SD, or median (interquartile range). *: Of the contralateral side

**Table 2:** T1 values of transverse carpal ligament (TCL), median nerve (MN), sheaths of the flexor carpi tendons (SFCT), subcutaneous fat (SCF), muscle of the thenar eminence (MTE), and global contouring of the wrist (GCW) of patients with cardiac amyloidosis (CA) and control subjects.

T1 Values (ms)	CA (n = 36)	Controls (n = 69)	*p*	Adjusted *p* *
TCL	829 (725–928)	994 (926–1085)	<0.001	0.002
MN	1234 (1130–1333)	1406 (1289–1528)	<0.001	0.04
SFCT	1030 (924–1122)	1175 (1104–1287)	<0.001	<0.001
SCF	989 (876–1142)	1165 (1014–1295)	0.003	0.02
MTE	185 (143–272)	205 (165–293)	0.40	0.26
GCW	934 (865–1025)	1111 (1029–1174)	<0.001	0.009

Data are median (interquartile range). * adjusted for age and sex.

## Data Availability

The data analyzed in the current study are available from the corresponding author upon reasonable request.

## Supplementary Materials

The following supporting information can be downloaded at: https://www.mdpi.com/article/10.3390/jcm14207374/s1. Table S1. Comparison of age and wrist tissue T1 values between patients with cardiac amyloidosis (CA) and control subjects older than 45 years.

## Abbreviations

The following abbreviations are used in this manuscript:

ATTR-CA	transthyretin cardiac amyloidosis
ECV	extracellular volume
GCW	global contouring of the wrist
HFpEF	heart failure with preserved ejection fraction
MN	median nerve
MTE	muscle of the thenar eminence
SCF	subcutaneous fat of the wrist
SFCT	sheaths of the flexor carpi tendons
TCL	transverse carpal ligament
