# Supplementary material for: Reduced Native T1 Values of Wrist Tissues in Transthyretin Cardiac Amyloidosis
Source: J Clin Med. 2025 Oct 18;14(20):7374. doi: 10.3390/jcm14207374 (PMC12565169; doi:10.3390/jcm14207374)
Supplement: Supplementary file 1 [file jcm-14-07374-s001.zip › jcm-3908016-supplementary.pdf]

Table S1. Comparison of age and wrist tissue T1 values between patients with cardiac amyloidosis (CA) and control subjects older than 45 years.

	**CA (n = 36)**	**Controls (n = 33)**	***p***
Age (years)	78 ± 9	54 ± 10	<0.001
$T1_{TCL}$ (ms)	829 (725–928)	1038 (911–1090)	<0.001
$T1_{MN}$ (ms)	1234 (1130–1333)	1390 (1289–1528)	<0.001
$T1_{SFCT}$ (ms)	1030 (924–1122)	1222 (1101–1287)	<0.001
$T1_{SCF}$ (ms)	989 (876–1142)	1200 (1008–1299)	0.006
$T1_{MTE}$ (ms)	185 (143–272)	277 (165–293)	0.60
$T1_{GCW}$ (ms)	934 (865–1025)	1075 (1029–1174)	<0.001
